# Can the Preoperative Serum Lactate Level Predict the Extent of Bowel Ischemia in Patients Presenting to the Emergency Department with Acute Mesenteric Ischemia?

**DOI:** 10.1155/2017/8038796

**Published:** 2017-02-05

**Authors:** Peter C. Ambe, Kai Kang, Marios Papadakis, Hubert Zirngibl

**Affiliations:** Department of Surgery II, HELIOS Universitätsklinikum Wuppertal, Witten-Herdecke University, Heusnerstr. 40, 42283 Wuppertal, Germany

## Abstract

*Purpose.* Early recognition of acute mesenteric ischemia (AMI) can be challenging. Extensive bowel necrosis secondary to AMI is associated with high rates of mortality. The aim of this study was to investigate the association between preoperative serum lactate level and the extent of bowel ischemia in patients with AMI.* Methods.* Data of patients with abdominal pain and elevated serum lactate undergoing emergency laparotomy for suspected AMI within 24 hours of presentation was retrospectively abstracted. The length of the ischemic bowel segment was compared with the preoperative serum lactate level.* Results.* 36 female and 39 male patients, with median age 73.1 ± 12.3 years, were included for analysis. The median preoperative lactate was 2.96 ± 2.59 mmol/l in patients with ≤50 cm, 6.86 ± 4.08 mmol/l in patients with 51–100 cm, 4.73 ± 2.76 mmol/l in patients with >100 cm ischemic bowel, and 14.07 ± 4.91 mmol/l in the group with multivisceral ischemia.* Conclusion.* Although elevated serum lactate might permit an early suspicion and thus influence the clinical decision-making with regard to prioritization of surgery in patients with suspected AMI, a linear relationship between serum lactate and the extent of bowel ischemia could not be established in this study.

## 1. Introduction

Acute mesenteric ischemia (AMI) is a life-threatening emergency with extremely high rates of mortality following occlusion of visceral vessels with bowel ischemia, infarction, and necrosis [1]. The outcome of AMI has not improved over the last decades despite advances in modern medicine [2–4]. This is mostly due to difficulties in recognizing the condition in an early stage prior to extensive bowel necrosis and multiple organ failure. Clinically, patients with AMI present with nonspecific abdominal complaints. Although physical examination might not distinguish AMI from other abdominal pathologies, discordance between findings on abdominal examination and the subjectively perceived severity of abdominal pain (by the patient) is commonly reported. Thus the preoperative diagnosis of AMI is based on a high degree of suspicion.

The level of lactate in the peripheral blood has been used as a possible marker for AMI [5–7]. Serum lactate has previously been described as a surrogate marker for disease severity and high lactate levels are thought to correspond with the severity of AMI [8–10]. While the debate about the diagnosis of AMI with the use of biomarkers is still ongoing, no data exists on the possible association between serum lactate level and the extent of bowel ischemia in patients with AMI. The aim of this study was to investigate the association between preoperative serum lactate level and the extent of bowel ischemia in patients undergoing emergency laparotomy for suspected AMI.

## 2. Methods

Data of patients undergoing emergency explorative laparotomy for suspected AMI within a five-year period from 2009 to 2014 in the department of surgery of a University Hospital was retrospectively abstracted. A written consent was received from each patient (or his/her legal representative) included in this study.

Patients were consecutively recruited after presenting to the emergency department with abdominal pain. All patients presenting to our emergency department are seen by an experienced emergency physician following a strict triage protocol. Patients are managed in an interdisciplinary setup. All cases with abdominal pain are seen by a gastroenterologist and a surgical consultant.

As part of our standards, samples are taken for blood chemistry including blood gas analysis (BGA) in all cases. Serum lactate was recorded following BGA. Lactate in this study represents the L-isomer (L-lactate), which is a product of human anaerobic metabolism.

Besides physical examination, ultrasound sonography, plain radiographs, or computed tomography (CT) scans of the abdomen are ordered as needed. Surgery is indicated based on the clinical impression of the surgical consultant or following confirmation of pathologic findings on abdominal imaging with the need of surgical intervention.

The study population included patients undergoing emergency or urgent laparotomy for suspected AMI. Two surgeons retrospectively reviewed our electronic operating room schedule and all consecutive cases undergoing emergency laparotomy for suspected mesenteric ischemia were selected. The data retrieved was crosschecked with the admission data and only patients undergoing surgery within 24 hours of admission were included. All cases undergoing surgery >24 h from the time of admission were excluded from this study. Demographic data including age, gender, and body mass index (BMI) were retrieved for each patient. The American Society of Anesthesiologist's (ASA) score was retrieved from the anesthesiology protocols. Only patients undergoing emergency exploratory or urgent laparotomy for suspected AMI with elevated serum lactate were included for analysis.

Surgery was performed as either an emergency procedure (next procedure) or an urgent procedure (same day procedure) depending on the clinical presentation and findings from abdominal imaging. Resuscitation with fluid, electrolyte, intravenous pain medication, and broad band antibiotics was initiated in the emergency department prior to surgery in all cases. The leading surgeon was an experienced surgical attending or a surgical fellow under direct supervision by an experienced attending.

A median laparotomy was performed in all cases. All intra-abdominal organs were exposed and inspected. The underlying pathology was identified during surgery. In cases with bowel ischemia, the visceral vessels were explored. If a primary vascular etiology was not evident (e.g., herniation), the underlying cause of bowel ischemia was addressed and the bowel was given some time to recover. Bowel resection was performed in cases with irreversible ischemia, infraction, or necrosis. The extent and location of bowel ischemia was documented. If extensive ischemia with multivisceral involvement (small bowel, large bowel, gallbladder, liver, spleen, kidney, etc.) was found during laparotomy no further intervention was performed due to the poor prognosis. The decision to perform a second look laparotomy was made on an individual basis. The abdomen was closed in all cases, independent of the need for a second look laparotomy. All patients were managed in the ICU.

The preoperative serum lactate level was retrieved from the preoperative BGA. The findings from contrast enhanced computed tomography with angiography (CT angiography) were recorded where available. The operative notes were consulted for information on the diagnosis during laparotomy. Besides, the extent of bowel involvement in centimeters (cm), the bowel segment involved, and the surgical procedures performed were retrieved in each case. The fate of the patient following surgery was gained from the final discharge notes.

Since our primary goal was to investigate the association between preoperative serum lactate and the extent of bowel ischemia, the study population was divided into subgroups depending on the length of the ischemic bowel segment using 50 cm as cut-off: ≤50 cm, 51–100 cm, >100 cm, and multivisceral. The median preoperative serum lactate values were compared with the length of the ischemic bowel segment.

The Statistical Package for Social Science (SPSS®), IBM, version 22, was employed to analyze the collected data. Because the data was not normally distributed, continuous variables were described using absolute case numbers and percentages, while central tendencies were described using medians with the corresponding interquartile ranges (±) with a 95% confidence interval. Statistical significance was calculated using Mann–Whitney *U* Test where necessary with the level of significance placed at *p* < 0.05. Finally, multivariate analysis was used to better study the association between preoperative serum lactate and the length of bowel ischemia.

The primary endpoint was the association between the level of preoperative lactate and the extent of bowel ischemia. The secondary endpoint was the rate of in-hospital mortality.

## 3. Results

Within the period of investigation 75 patients presenting to the emergency department with acute abdominal pain and elevated serum lactate underwent surgical exploration for suspected AMI. The baseline characteristics of the study population are presented in Table 1.

Cardiovascular disorders including atrial fibrillation, hypertension, ischemic heart disease, and peripheral artery disease were the most common concomitant disorders in this study and were recorded in 55 cases (69%). Type 2 diabetes was present in 20 cases (27%). Chronic obstructive pulmonary disease was present in nine cases (12%) while chronic kidney disease was seen in 12 cases (16%).

**Figure 1:**
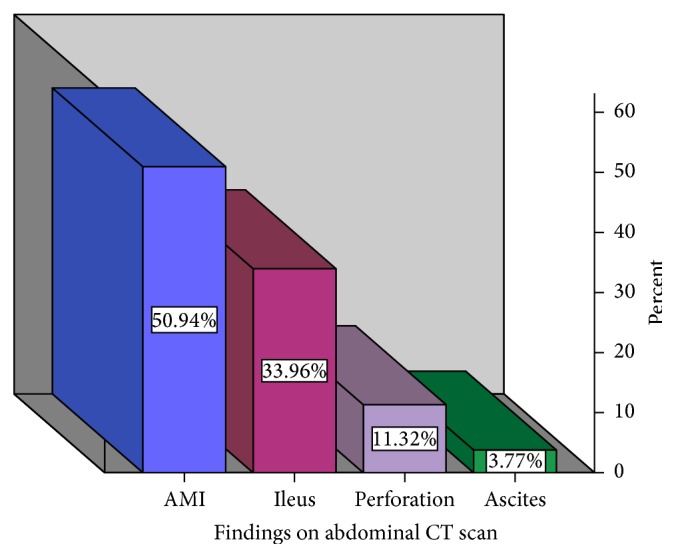
Preoperative CT findings. AMI was confirmed in just about 50% of cases following CT angiography. CT: computed tomography. Multiple findings were present in 40% of cases.

Ultrasound sonography was performed in all cases, plain radiographs of the abdomen were obtained from 15 patients (20.0%), while CT angiography was ordered in 56 cases (74.7%). The preoperative CT findings are presented in Figure 1. More than one pathologic finding was seen in 45 patients (60%) following CT.

The cause of AMI was occlusive-embolic in 32 cases (42.6%), occlusive thrombotic in 18 cases (24%), and nonocclusive in 13 cases (17.3%). Bowel ischemia was absent in 12 cases (16%). Ischemia was limited to the small bowel in 31 cases (41.3%). The ileum and the right colon were involved in 13 cases (17.3%) while the left colon was involved in 12 cases (16.0). The length of the ischemic bowel segment was ≤50 cm in 29 cases (38.7%), 51–100 cm in 13 cases (17.3%), and >100 cm in 15 cases (20%). Multivisceral ischemia involving the bowel, gallbladder, and liver was seen in seven cases (9.3%).

Segmental resection of the small bowel with anastomosis was performed in 18 cases (24.0%); segmental resection of the ileum and the right colon with anastomosis was done in 16 cases (21.3%); segmental resection of the left colon with anastomosis was performed in seven cases (9.3%), while stoma creation was performed in 15 cases (20.0%). Bowel resection was not performed in 19 cases (25.3%) including 12 cases without bowel ischemia and seven cases with multivisceral ischemia.

**Figure 2:**
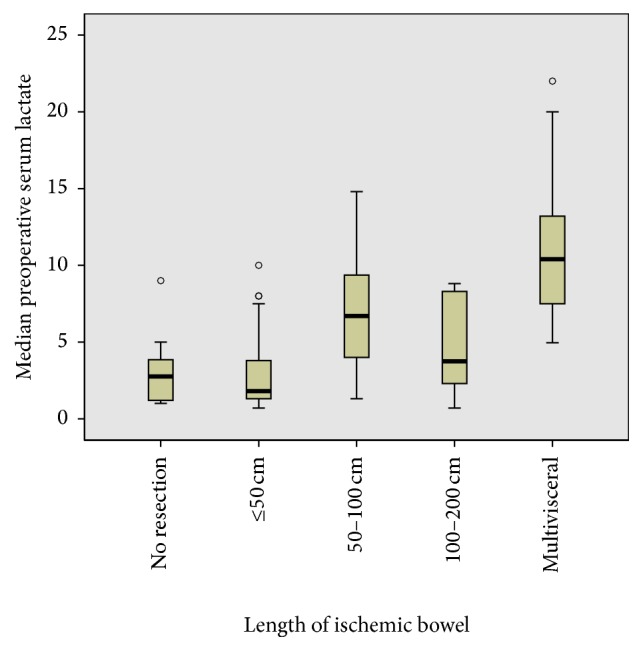
Median preoperative serum lactate. There was no lineal association between the preoperative lactate level and the extent of bowel ischemia.

The median preoperative serum lactate was 2.96 ± 2.59 mmol/l in the group with ≤50 cm bowel ischemia, 6.86 ± 4.08 mmol/l in the group with 51–100 cm, 4.73 ± 2.76 mmol/l in the group with >100 cm, and 14.07 ± 4.91 mmol/l in the group with multivisceral ischemia. Generally, no linear association was seen between the median preoperative serum lactate levels and the length of ischemic bowel (Figure 2). However, the median preoperative serum lactate level was significantly higher in the group with multivisceral ischemia. This trend was supported by the results of multivariate analysis.

**Figure 3:**
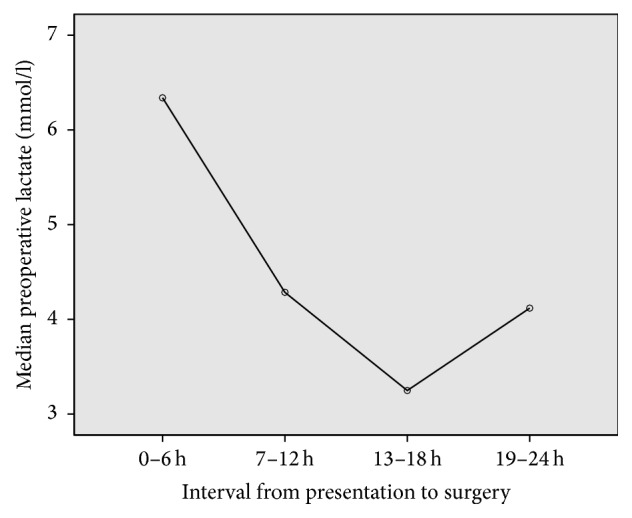
Surgical scheduling. Surgery was prioritized in patients with high preoperative lactate values.

**Figure 4:**
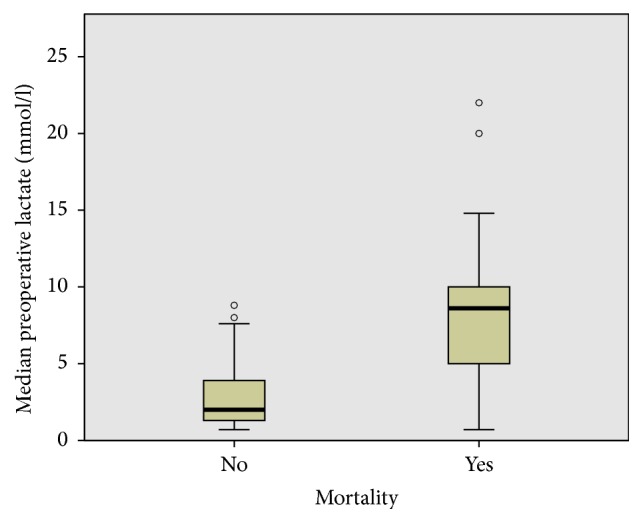
Mortality. High lactate values correlated significantly with mortality.

Figure 3 shows the relationship between preoperative lactate level and the time interval to surgery. All patients were managed in the intensive care unit following surgery. A second look was performed in 11 cases (14.7%) including four cases after embolectomy, five after small bowel resection, and two after large bowel resection. The rate of mortality was 40.0% (30 deaths). The cause of death was multiple organ failure in all cases. There was a statistically significant association between the median preoperative lactate and death (8.4 ± 4.9 versus 2.9 ± 2.1, *p* = 0.001), Figure 4.

## 4. Discussion

The aim of this retrospective analysis was to investigate the association between preoperative serum lactate and the extent of bowel ischemia in patients undergoing explorative laparotomy for suspected AMI. Seventy-five consecutive patients with suspected AMI undergoing emergency or urgent laparotomy within 24 h of presentation in the emergency department were included for analysis. The median preoperative serum lactate levels were compared with the length of ischemic bowel segment following laparotomy. Low preoperative lactate values were recorded in cases with limited or no bowel ischemia while high lactate levels were seen in patients with multivisceral ischemia. However, no linear association could be established between preoperative serum lactate level and the length of ischemic bowel segment. This trend was confirmed on multivariate analysis. High lactate values were associated significantly with risk of death.

Bowel ischemia with infarction and necrosis following AMI is a true surgical emergency. Acute mesenteric ischemia has for many decades been associated with high rates of mortality and delayed intervention secondary to missed diagnosis is the most common reason for this extremely poor outcome. Symptoms of AMI could be nonspecific or vague with a poor correlation between clinical presentation and findings from abdominal examination. Despite extensive research no sensitive marker has been established so far. Thus the debate about a sensitive diagnostic marker for AMI still goes on.

Presently, CT angiography represents the best diagnostic modality for patients with suspected AMI. However, the sensitivity to CT angiography in detecting AMI is estimated at just 64% [11]. In our series, AMI was confirmed on CT angiography in just 51% of cases. Besides CT angiography, serum lactate has been suggested as a diagnostic marker for AMI [11].

In the present study, low serum lactate values were recorded in cases without bowel ischemia and in those with limited bowel involvement (≤50 cm) while high values were seen in patients with multivisceral involvement. However, the median preoperative serum lactate level was lower in the group with >100 cm of ischemic bowel compared to the group with 50–100 cm ischemic bowel. While this trend might be due to the small number of cases included in the group with >100 cm ischemic bowel, this finding suggests that the relationship between preoperative serum lactate level and the extent bowel ischemia might not be linear. This result is in accordance with data from a recently published retrospective analysis by Studer et al. [12], who found no significant association between serial lactate measurements and the length of the ischemic bowel segment.

Negative laparotomy with regard to AMI was performed in 16% of cases. Although the indication for laparotomy was suspected AMI, bowel ischemia could not be confirmed in these patients during laparotomy. This was a rather surprising finding since only patients with elevated serum lactate and suspected AMI were included in this study. This finding limits the diagnostic value of serum lactate as a surrogate marker for bowel ischemia.

An intriguing finding in this study was the effect of preoperative serum lactate on surgical decision-making with regard to scheduling of surgery. Surgical priority was given to patients with high serum lactate values in comparison to those with lower values. Significantly more patients with high lactate values underwent emergency laparotomy within six hours of presentation. Extensive bowel involvement and multivisceral ischemia was evident during laparotomy in a significant majority of these cases. As expected, the outcome was fatal in all cases with multivisceral ischemia.

The rate of mortality in this series was 40%. This was similar to the rate reported by Stuber et al. [12] but below the 60–80% rates reported elsewhere [2, 9, 13, 14]. Although one may argue that the high rates of mortality recorded in this and other series might be primarily due to serious concomitant medical conditions and not to AMI per se [13, 15, 16], high levels of preoperative serum lactate and the extent of visceral involvement were associated with significantly higher mortality. All cases of mortality were recorded during the postoperative period in the ICU and multiple organ failure was the cause of death in all cases. This finding is in accordance with existing data [17]. The lower rate of mortality recorded in this series in comparison to the 60–80% rate reported in other studies could only be achieved through an early diagnosis and intervention.

Although our results failed to confirm a linear association between the level of preoperative serum lactate and the extent of bowel ischemia in patients with AMI, elevated serum lactate values might have enabled an early suspicion of AMI. Besides, high lactate levels significantly influenced clinical decision-making with respect to prioritization of surgery. As expected, high serum lactate value was associated with high rate of mortality.

## 5. Limitations

Serum lactate elevation could be secondary to a number of conditions including ischemia and necrosis of visceral organs, hepatic dysfunction with impaired metabolism, and hypotension with impaired clearance. Besides, it is well recognized that serum lactate elevation may not be present in a significant portion of patients with AMI. The results reported in this study are based on a rigid selection of patients with a high probability of bowel ischemia after excluding other confounding conditions. More so, only patients presenting with acute onset of abdominal pain and elevated serum lactate were included in this study. Thus the results recorded in this series are based on a strict patient selection, constituting a considerable selection bias. Besides, only patients managed within 24 hours of presentation were included for analysis. Thus the results from this study cannot be projected on patients with long standing symptoms >24 hours. The retrospective study design also must be seen as a limitation. Data on the degree of the color change induced by ischemia that might have enabled the use of a universal color index scale was not available. The lack of a control group, the size of the study population, and the retrospective study design represent major limitations in this study.

## 6. Conclusion

Although elevated lactate might permit an early suspicion and thus influence the clinical decision-making with regard to prioritization of surgery in patients with AMI, a linear association between serum lactate and the extent of bowel ischemia could not be established in this study.

## Figures and Tables

**Table 1:** Baseline characteristics. ASA: American Society of Anesthesiologists. Yrs: years. ±: interquartile range.

Demographic characteristics	
Gender	
(i) Female	36 (48.0%)
(ii) Male	39 (52.0%)
Age	
(i) Median	73.1 ± 12.3 yrs
(ii) Range	29–92 yrs
ASA Score	
(i) 1	1 (1.3%)
(ii) 2	11 (14.7%)
(iii) 3	28 (37.3%)
(iv) 4	28 (37.3%)
(v) 5	7 (9.3%)
